# Exploring the Therapeutic Potential of Natural Compounds and Plant Extracts in Human Health

**DOI:** 10.3390/biom15060774

**Published:** 2025-05-27

**Authors:** Laura Zeppa, Cristina Aguzzi, Maria Beatrice Morelli

**Affiliations:** 1 School of Biosciences and Veterinary Medicine, University of Camerino, 62032 Camerino, Italy; laura.zeppa@unicam.it; 2 School of Pharmacy, University of Camerino, 62032 Camerino, Italy; cristina.aguzzi@unicam.it

A growing body of evidence supports the various potential advantages of plant extracts and plant extract-based products in promoting human health and addressing a broad range of pathological conditions [1]. Over the past decades, scientific interest in natural compounds has surged, driven by the urgent need for safer, more sustainable, and cost-effective therapeutic alternatives. These bioactive compounds, often derived from plants used in traditional medicine, represent a vast and largely untapped reservoir of pharmacological agents with multifaceted biological activities [2].

Natural products are characterized by their chemical diversity and ability to modulate multiple molecular targets simultaneously, making them especially valuable in the treatment of complex diseases with multifactorial pathogenesis, such as cancer, cardiovascular disorders, metabolic syndromes, and neurodegenerative diseases [3,4,5]. Furthermore, many of these compounds exhibit favorable safety profiles, often accompanied by antioxidant, anti-inflammatory, immunomodulatory, and antimicrobial properties.

Advances in biotechnology, phytochemistry, and molecular pharmacology have significantly improved our ability to isolate, characterize, and understand the mechanisms of action of these natural compounds [6,7]. This has enabled not only the identification of novel drug leads but also the development of standardized extracts and nutraceutical formulations aimed at health promotion and disease prevention.

In this context, the integration of natural compounds into modern therapeutic strategies offers promising avenues for innovation in both preventive and personalized medicine. The present Special Issue aims to provide an overview of recent progress in this field, highlighting experimental, review, and clinical studies that explore the therapeutic potential of plant-derived molecules in diverse pathological contexts.

Three original studies explored the cardiovascular effects of different plant-derived compounds. Fernando et al. investigated phloridzin docosahexaenoate (PZ-DHA), an ω-3 fatty acid ester derived from a flavonoid precursor, demonstrating its anti-angiogenic properties in both in vitro and in vivo models [8,9]. PZ-DHA significantly inhibited endothelial cell proliferation, migration, and tubule formation, while downregulating the Akt signaling pathway and VEGF165-induced RhoA activation (contribution 1). Emodin, a naturally occurring anthraquinone, was shown to mitigate pathological cardiac hypertrophy in hypertensive mice while also alleviating gut microbiota alterations associated with hypertension (contribution 2) [10] and contributing to the improvement of cardiac diseases in general [11,12]. Similarly, Obeidat et al. examined (+)-taxifolin (TAX), a flavonoid with antioxidant and anti-inflammatory properties, in a murine model of isoproterenol-induced acute myocardial injury. TAX treatment significantly reduced myocardial damage, oxidative stress, and inflammation, primarily through upregulation of the Nrf2/HO-1 pathway (contribution 3) [13].

Beyond cardiovascular implications, natural compounds also hold promise in addressing skeletal and joint disorders. Hecht et al. demonstrated that early postnatal administration of resveratrol preserved joint integrity and reduced pain in a mouse model of pseudoachondroplasia—a condition linked to mutations in the cartilage oligomeric matrix protein (contribution 4) [14].

The anti-inflammatory properties of natural compounds in the context of obesity were the focus of two complementary studies. Carpi et al. explored tanshinone IIA and cryptotanshinone, diterpenoids isolated from *Salvia miltiorrhiza* Bunge, in inflamed human adipocytes [15,16,17], where both compounds reduced proinflammatory cytokine and chemokine release, as well as monocyte recruitment (contribution 5). A review by Ngamsamer et al. addressed the beneficial roles of anthocyanins, water-soluble flavonoid pigments abundant in fruits and vegetables. These compounds have strong antioxidant effects, reducing reactive oxygen species and mitigating chronic inflammation [18,19]. Moreover, they were shown to regulate gut dysbiosis associated with obesity and to downregulate inflammatory cytokine production in both in vitro and in vivo models (contribution 6).

Several natural compounds also influence metabolic pathways and redox balance. In particular, plant-derived molecules are involved in the regulation of Nrf2, a transcription factor critical in oxidative stress responses and metabolic regulation. Moreover, natural products can stimulate heme oxygenase (HMOX) activity, an enzyme responsible for heme catabolism. This pathway leads to the production of bilirubin, a molecule with emerging roles in cytoprotection, antioxidative defense, and metabolic homeostasis. Flavonoids, curcumin, astragaloside, and vitamins are some of the natural products involved in the modulation of bilirubin and the metabolic Nrf-2 pathway. Other natural compounds can mimic the products of heme degradation or modulate this pathway directly, suggesting further therapeutic potential (contribution 7).

Cancer is another key area where natural compounds show promising effects. Ellagic acid, a polyphenol present in various fruits and nuts, has demonstrated anticancer activity by modulating numerous hallmarks of cancer—including cell proliferation, apoptosis, angiogenesis, and immune evasion [20]. A comprehensive review by Čižmáriková et al. details its mechanisms in cancer prevention and treatment (contribution 8). Another review focused on the synergistic use of polyphenols—both flavonoid and non-flavonoid types—with bortezomib (BTZ), a first-line proteasome inhibitor for multiple myeloma [21]. A total of nine flavonoids (e.g., icariin, EGCG, morin) and four non-flavonoids (e.g., resveratrol, curcumin) were identified as enhancing BTZ’s anticancer effects, underscoring the role of dietary components in augmenting conventional chemotherapy (contribution 9).

Some natural products exhibit a broad spectrum of biological activities. A review by Tematio Fouedjou et al. presented the therapeutic properties of various Cordyline species, traditionally used in folk medicine. These plants demonstrated antimicrobial, antiparasitic, antioxidant, antiproliferative, and organ-protective effects (contribution 10). Similarly, date seeds (*Phoenix dactylifera* L.) were highlighted for their high phenolic content and diverse pharmacological actions, including anti-inflammatory, antioxidant, antibacterial, antiviral, antidiabetic, and anticancer effects [22,23] (contribution 11).

Lastly, the clinical potential of natural compounds has been investigated in human studies. Al-Jubori et al. compiled clinical trials assessing different species of gum Arabic in the management of diseases such as sickle cell anemia, rheumatoid arthritis, periodontitis, metabolic and gastrointestinal disorders, and kidney diseases. These results highlight the transition of natural compounds from traditional uses to evidence-based applications in clinical settings (contribution 12).

In conclusion, the studies presented in this Special Issue collectively reinforce the therapeutic promise of natural compounds and plant-based products across a wide range of diseases. Continued research into their mechanisms of action, synergistic potential with standard therapies, and clinical translation will further validate their role in modern medicine.

## Abbreviations

The following abbreviations are used in this manuscript:

PZ-DHA	phloridzin docosahexaenoate
TAX	taxifolin
HMOX	heme oxygenase
BTZ	bortezomib
